# Targeting Mortalin by Embelin Causes Activation of Tumor Suppressor p53 and Deactivation of Metastatic Signaling in Human Breast Cancer Cells

**DOI:** 10.1371/journal.pone.0138192

**Published:** 2015-09-16

**Authors:** Nupur Nigam, Abhinav Grover, Sukriti Goyal, Shashank P. Katiyar, Priyanshu Bhargava, Pi-Chao Wang, Durai Sundar, Sunil C. Kaul, Renu Wadhwa

**Affiliations:** 1 Drug Discovery and Assets Innovation Lab, DBT-AIST International Laboratory for Advanced Biomedicine (DAILAB), Biomedical Research Institute, National Institute of Advanced Industrial Science & Technology (AIST), Tsukuba, Japan; 2 Graduate School of Life and Environmental Sciences, University of Tsukuba, Tsukuba, Japan; 3 School of Biotechnology, Jawaharlal Nehru University, New Delhi, India; 4 Department of Biochemical Engineering & Biotechnology, Indian Institute of Technology (IIT) Delhi, New Delhi, India; Kyung Hee University, KOREA, REPUBLIC OF

## Abstract

Embelin, a natural quinone found in the fruits of *Embelia ribes*, is commonly used in Ayurvedic home medicine for a variety of therapeutic potentials including anti-inflammation, anti-fever, anti-bacteria and anti-cancer. Molecular mechanisms of these activities and cellular targets have not been clarified to-date. We demonstrate that the embelin inhibits mortalin-p53 interactions, and activates p53 protein in tumor cells. We provide bioinformatics, molecular docking and experimental evidence to the binding affinity of embelin with mortalin and p53. Binding of embelin with mortalin/p53 abrogates their complex resulted in nuclear translocation and transcriptional activation function of p53 causing growth arrest in cancer cells. Furthermore, analyses of growth factors and metastatic signaling using antibody membrane array revealed their downregulation in embelin-treated cells. We also found that the embelin causes transcriptional attenuation of mortalin and several other proteins involved in metastatic signaling in cancer cells. Based on these molecular dynamics and experimental data, it is concluded that the anticancer activity of embelin involves targeting of mortalin, activation of p53 and inactivation of metastatic signaling.

## Introduction

Embelin (2,5-dihydroxy-3-undecyl-2, 5-cyclohexadiene-1, 4-benzoquinone), a naturally occurring benzoquinone, is a phenolic compound present in fruits of *Embelia ribes* Burm. f. (Primulaceae), commonly used in Indian traditional home medicine system, Ayurveda. Laboratory studies have identified its several pharmacological and therapeutic properties that have extended its use to treat fever, inflammatory diseases, mental disorders and several other pathological conditions including, bacterial infections, diabetes, myocardial injury and cancer [1, 2–7]. The anticancer activity of embelin is mediated by inhibition of X-linked anti-apoptotic protein (XIAP), NF-kappaB, PCAF, TACE, TNF-α and other cytokines [8–12]. Drug resistance of non-small cell lung cancer cells (NSCLC) caused by overexpression of XIAP was inhibited by embelin with an efficacy similar to XIAP siRNA. These caused activation of caspase 3-induced apoptosis suggesting the inhibition of XIAP by embelin as a therapeutic strategy for cisplatin-resistant NSCLC cells [13]. In addition to the anti-mitotic and apoptotic activities of embelin, it was shown to inhibit wound healing, single cell migration and endothelial ring formation in egg yolk assays for cancer cells metastasis and angiogenesis [2, 10]. However, the mechanisms of these activities remain unclear to-date. We had earlier reported embelin-induced inhibition of TACE and metastatic signaling proteins including MMPs, VEGF and hnRNP-K that causes malignant transformation of breast cancer cells [10].

Chemical structure of embelin is similar to natural coenzyme, Q10. Several studies have assigned medicinal properties of embelin to its free radical scavenging and anti-oxidant activities [14]. It was shown to inhibit lipid peroxidation and restore impaired Mn-superoxide dismutase in rat liver mitochondria [15]. Increase in pancreatic anti-oxidant enzymes, including superoxide dismutase, catalase and glutathione peroxidase and decrease in the thiobarbituric acid reactive oxygen species contents, was reported in streptozotocin-induced rat diabetes model. Based on such anti-oxidative potential of embelin, it was suggested to be useful for therapy of severe hyperglycemia [16]. Several studies have indicated that embelin may cause depolarization of mitochondrial membrane potential, uncoupling of electron transport chain and inhibit oxidative phosphorylation resulting in release of mitochondrial cytochrome C and activation of caspases triggering apoptosis [17–20].

Mortalin, a stress chaperone, is enriched in cancers [21–25]. It has multiple functions contributing to continued proliferation of cancer cells. These include mitochondrial-biogenesis, ATP production, anti-apoptosis, chaperoning, inactivation of tumor suppressor p53 and PI3K/AKT activities [26, 27]. Targeting mortalin by siRNA, ribozymes and small molecules including MKT-077 and Withaferin A resulted in growth arrest/apoptosis of cancer cells [28–34]. In light of the information that mortalin is a mitochondrial stress chaperone involved in carcinogenesis and metastasis, and embelin causes changes in the mitochondrial membrane potential of cells [17–20], the present study was planned to investigate the effect of embelin on mortalin and its impact on cancer cell properties. We found that embelin targets mortalin resulting in (i) nuclear translocation and reactivation of transcriptional activation function of p53 and (ii) downregulation of metastasis signaling proteins.

## Materials and Methods

### Cell culture, treatments and viability assays

Human breast cancer cells, MCF7 and MDA-MB-231, were obtained from Japanese Collection of Research Bioresources (JCRB, Japan) and cultured in DMEM (Life Technologies, Carlsbad, CA, USA)-supplemented with 10% fetal bovine serum and antibiotics at 5% CO_2_ and 95% air in a humidified incubator. Embelin (99% purity) was procured from (Sigma-Alrich, Japan) and was dissolved in DMSO to obtain 10 mM stock. Working concentrations (10–20 μM) were prepared in DMEM. Cells were treated with embelin at about 60–70% confluency. Equal volume of DMSO was used as a solvent control for untreated cells in all the assays. Morphological observations were taken using a phase contrast microscope (Nikon Eclipse TE300). Cell viability was determined by MTT assay (Life technologies, Carlsbad, CA, USA) following manufacturer’s instructions and as described earlier [10]. Briefly, cells after the treatment with embelin for 24–48 h were incubated with MTT (0.5 mg/mL) for 4 h followed by replacement of MTT- containing medium with 100 μL DMSO to dissolve formazan crystals. Absorbance was measured at 550 nm using a spectrophotometer (Tecan, Switzerland). For long-term viability, cells (500/well) were plated in 6-well dish, incubated to develop colonies for the next 10–15 days with a regular change in media (control or embelin-supplemented) every alternate day. Colonies were fixed in methanol, stained with 0.1% crystal violet, photographed and counted. Statistical significance of the data, obtained from three independent experiments, was calculated by QuickCals t-test calculator (GraphPad Software, Inc., CA).

### Cell cycle analysis by flow cytometry

Cells, seeded in 6-well dish, were treated with embelin (10 μM) for 48 h followed by harvesting by trypsinization. Cells were washed once with PBS and then fixed in ethanol. Fixed cells were washed with PBS and incubated first in RNase A for 1 h followed by incubation with Guava® cell cycle reagent (Merck Millipore, MA, USA) for 30 min. Flow Cytometry was performed using Guava PCA-96 System (Guava Technologies, Merck Millipore, MA, USA).

### Immunoblotting

Control and embelin-treated cells were harvested and lysed using RIPA (Radio Immune Precipitation Assay) buffer (Thermo Scientific, MA). Protein lysate (20 μg) was resolved in SDS-polyacrylamide gels, transferred to PVDF membrane and then probed with antibodies specific to mortalin, p53 (Santa Cruz), Bcl-2 (Cell Signaling Technologies Inc., MA, USA) and PARP-1 (Santa Cruz Biotechnology Inc., Texas, USA) followed by incubation with the respective secondary antibodies. Membranes were probed with anti β-actin antibody (Abcam) as an internal loading control. The protein bands were quantitated using ImageJ software (NIH, MA). Statistical significance of the data, obtained from three independent experiments, was calculated by QuickCals t-test calculator (GraphPad Software, Inc., CA).

### Immunostaining

Cells were cultured on coverslips. Embelin (10 μM, 24–48 h) was added when cells had attached well to the substratum. Following the treatment, cells were washed 2–3 times with cold 1 X PBS and fixed in methanol:acetone (1:1) for 5 min. Fixed cells were washed twice with 1 X PBS, permeabilized using 0.5% Triton X-100 in PBS for 10 min and blocked using 2% BSA in PBS for 15 min. Coverslips containing cells were then incubated with either anti-mortalin, -p53, -MMP-3, -MMP-9, -vimentin, -β-catenin or -TGF-β (Santa Cruz Biotechnology Inc., Texas) antibody for 2 h at room temperature, washed thrice with 0.2% Triton X-100 in PBS (TPBS) and incubated with Alexa Fluor conjugated respective secondary antibodies. After 3–6 washings with TPBS, coverslips were mounted and visualized under Carl Zeiss microscope (Axiovert 200 M).

### p53 dependent reporter assay

MCF7 cells, cultured in 6-well plates, were transfected with p53 reporter plasmid (PG13Luc) containing luciferase reporter driven by 13 repeats of p53 binding consensus sequence (a kind gift from the laboratory of Bert Vogelstein) using X-tremeGENE 9 DNA Transfection Reagent (Roche, BASEL, Switzerland). After overnight incubation, cells were treated with embelin (10 μM) for 24 h, washed with PBS and luciferase reporter assay was performed using Promega Dual-luciferase reporter assay kit (Promega, Madison, WI, USA). Cells were lysed by shaking-incubation in passive lysis for 15 min at room temperature. The lysate was then transferred to 96-well plate and incubated with LARII (Luciferase Assay Substrate) to measure the firefly luciferase activity. This was followed by immediate incubation of the samples with Stop & Glo Reagent to measure renilla luciferase activity using Promega Dual-luciferase reporter assay kit (Promega, Madison, WI, USA) and a spectrophotometer (Tecan, Switzerland).

### Detection of ROS (Reactive Oxygen Species)

Cells were seeded on coverslips placed in 12-well dish. After overnight culture, they were treated with either control or embelin (10 μM) containing medium for 48 h, followed by gentle washing with warm PBS. Cells were then incubated with carboxy-H_2_DCFDA provided in Image-iT^TM^ LIVE green reactive oxygen species detection kit (Molecular Probes, Eugene, OR). Cells were then washed thrice with warm PBS, mounted, and immediately imaged under Carl Zeiss microscope (Axiovert 200 M).

### Growth factor and metastasis proteins—Antibody membrane array

Conditioned media from control and embelin-treated cells were collected and centrifuged at 3000 rpm to remove cell debris. Clear supernatant was used to incubate the Human Growth Factor Antibody Array—membrane (Abcam, Cambridge, UK) following manufacturer’s instructions. Briefly, each membrane was first blocked by blocking buffer for 30 min at RT, followed by incubation with the conditioned medium for 2 h. Membranes were then sequentially washed with Wash buffer I and II (3 times, 5 min each with each buffer), incubated with biotin-conjugated anti-cytokines for 2 h, washed with Wash buffers I and II (3 times, 5 min each) and then incubated with HRP-conjugated streptavidin for 2 h at room temperature. The membranes were washed again using Wash buffers I and II (3 times, 5 min each), and chemiluminescence signals were detected with a mixture of buffer C and D in equal volume (1:1). The spot signal density was quantitated using ImageJ software (NIH, MA, USA). All the reagents (Blocking buffer, Wash buffers I and II, Biotin-conjugated anti-cytokines, HRP-conjugated streptavidin and detection buffers C and D) were supplied in the Human Growth Factor Antibody Array—Membrane kit (Abcam, Cambridge, UK).

### Pathway analysis

PathVisio 3.1.3 was used to study the biological pathways involving target genes identified by antibody membrane array analysis of the conditioned media, obtained from control and embelin-treated cells. *Wikipathways_Homo_sapiens_Curation-Analysis Collection* and *wikipathways_Homo_sapiens_Curation-Reactome_Approved* were used to identify the pathways with gene mapping database *Hs*_*derby*_*20130701*.*bridge* [35, 36].

### Quantitative Real—Time Polymerase Chain Reaction (qRT-PCR)

Total RNA was extracted from control and embelin-treated cells using Qiagen RNeasy kit (Qiagen, Limburg, Netherlands). Total RNA (2 μg) was reverse transcribed to cDNA using the QuantiTect Reverse Transcriptase kit (Qiagen, Limburg, Netherlands) following manufacturer’s protocol. Briefly, total extracted RNA was incubated with genomic DNA Wipeout buffer and RNase-free water at 42°C for 2 min, followed by addition of Quantiscript reverse transcriptase (RT), Quantiscript RT buffer and RT Primer mix. The mixture was incubated at 42°C for 15 min, and then at 95°C for 3 min. Quantitative Real-Time PCR amplifications were performed using equal amount of synthesized cDNA with gene specific sense and antisense primer sets (as detailed in Table 1) using SYBR Select Master Mix (Life Technologies, Carlsbad, CA, USA). Analyses of the PCR reaction products were performed using the relative analysis 2^-ΔΔC^T method [37].

**Table 1:** Sequence of primers used for Quantitative Real-Time PCR.

Target Gene	Primer	Sequence
**Mortalin**	Sense	5′- AGCTGGAATGGCCTTAGTCAT-3′
	Antisense	5′- CAGGAGTTGGTAGTACCCAAATC -3′
**Wnt-3a**	Sense	5′- CAAGATTGGATCCAGGAGT -3′
	Antisense	5′- TCCCTGGTAGCTTTGTCCAG-3′
**MMP-9**	Sense	5′-TGTACCGCTATGGTTACACTCG -3′
	Antisense	5′- GGCAGGGACAGTTGCTTCT -3′
**MMP-3**	Sense	5′- CTGGACTCCCGACACTCTGGA -3′
	Antisense	5′- CAGGAAAGGTTCTGAAGTGACC-3′
**β-catenin**	Sense	5′- AAAGCGGCTGTTAGTCACTGG -3′
	Antisense	5′- GACTTGGGAGGTATCCACATCC -3′
**TGF- β**	Sense	5′- CAATTCCTGGCGATACCTCAG-3′
	Antisense	5′- GCACAACTCCGGTGACATCAA-3′
**Vimentin**	Sense	5′- CCTTGAACGCAAAGTGGAATC-3′
	Antisense	5′- GACATGCTGTTCCTGAATCTGAG -3′
**18s (internal control)**	Sense	5′-CAGGGTTCGATTCCGTAGAG -3′
	Antisense	5′-CCTCCAGTGGATCCTCGTTA—3′

### Statistical analysis

Quantitation of data was performed using ImageJ software (NIH, MA). Statistical significance of the data from three sets of experiments was calculated by QuickCals t-test calculator (GraphPad Software, Inc., CA).

### Docking of mortalin and p53 with embelin

Crystal structures of human mortalin (PDB ID: 4KBO) and p53 (PDB ID: 1OLG), used as targets in molecular docking studies, were obtained from PDB (Protein Data Bank) [38]. The structure of ligand embelin (compound ID: 3218) was obtained from PubChem compound database [39]. The docking studies were performed using ArgusLab 4.0.1 {M T: ArgusLab 4.0.1. Planaria software. In. Seattle, W.A.: LLC; 2004}. The proteins and the ligand molecules were prepared and the binding sites were defined using the selected residues of the receptors. The grid resolution was kept at 0.4 Å and an exhaustive search docking was performed. The docking process was carried out using “ArgusDock” docking engine, calculation type as “Dock” and the ligand was kept in the flexible mode. The scoring function used was an empirical scoring function “AScore” which takes into account van der Waals energy, hydrophobic component, hydrogen bonds and deformation penalty. The parameter file “AScore.prm” was used to compute the binding energies. A total of 150 docking poses were generated and ranked according to the scoring function and the highest scoring pose was used in the further study.

### Molecular dynamics simulations of embelin-docked mortalin complex

MD simulations were carried out using the GROMACS package [40, 41]. The force field Gromos43a1 [42] was used for both embelin-docked mortalin and p53 complexes [40]. The GROMACS topology file was generated using antechamber python parser interface (ACPYPE) script. The docked protein structure was solvated in a cubic box and the water molecules and appropriate counter-ions were added to neutralise the system. The solvated system was minimized using steepest descent and conjugate gradient methods until the force on each atom was less than 100 kJ/mol/nm. These geometry minimized systems were used for 10 ns for carrying out isobaric (constant pressure-temperature) MD simulations. The temperature and pressure of the system was maintained at 300 K and 1 atmosphere pressure respectively with a time constant of 5 ps. A 2-fs time step was used for integrating the equations of motion. Particle Mesh Ewald summation method along with periodic boundary conditions were also applied throughout to calculate the electrostatic potential between partial charges on atoms. In order to resolve the specificity of binding, molecular dynamic analysis was also performed with mutated mortalin in which its crticial residues binding with embelin were mutated to alanine.

## Results

### Embelin downregulates mortalin and causes growth arrest of cancer cells

**Fig 1:**
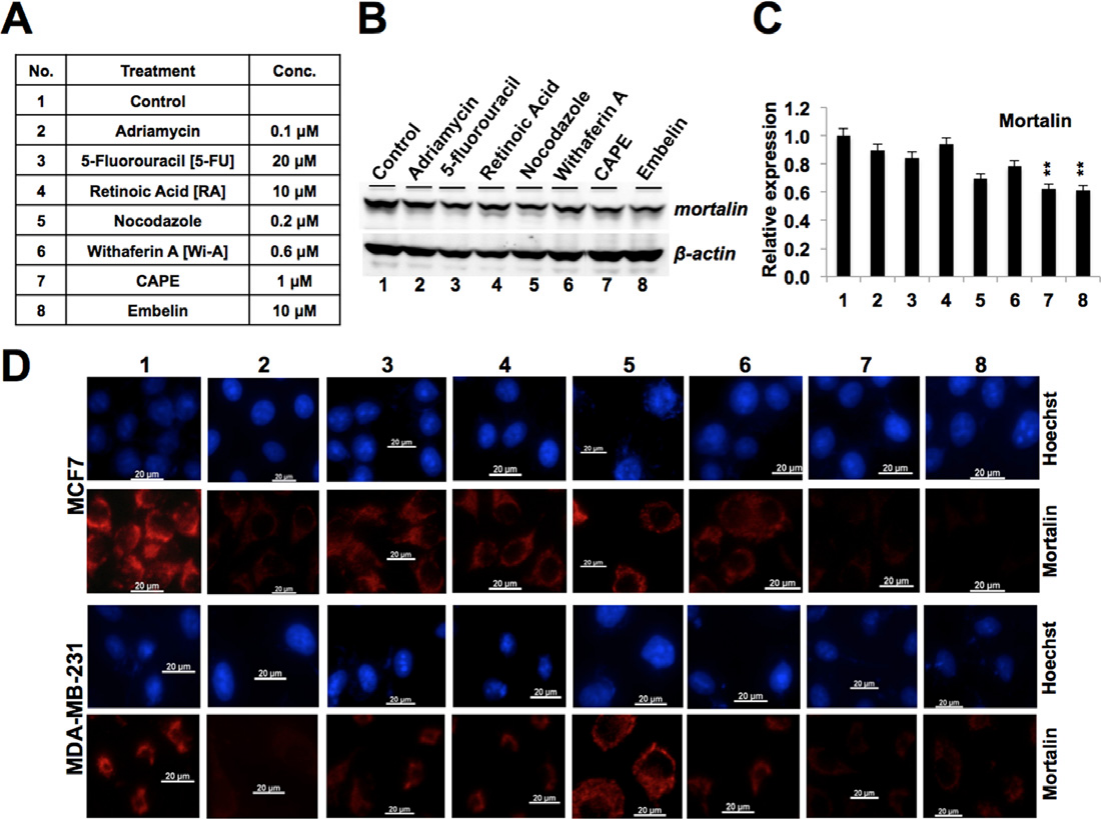
Effect of anticancer drugs on mortalin expression. (**A**) Concentrations of different anticancer drugs used to treat human breast cancer cell lines (MCF7 and MDA-MB-231). (**B**) Expression levels of mortalin in control and treated cells as detected by Western blotting. Actin was used as an internal control. (**C**) Quantitation of mortalin expression level obtained in Western blotting. Quantitation was performed using ImageJ software, and statistical significance was calculated by QuickCals t-test calculator (GraphPad Software Inc., CA) (**p<0.01). (**D**) Immunostaining of mortalin in control and treated breast cancer cells (MCF7 and MDA-MB-231); nuclei were stained with Hoechst.

**Fig 2:**
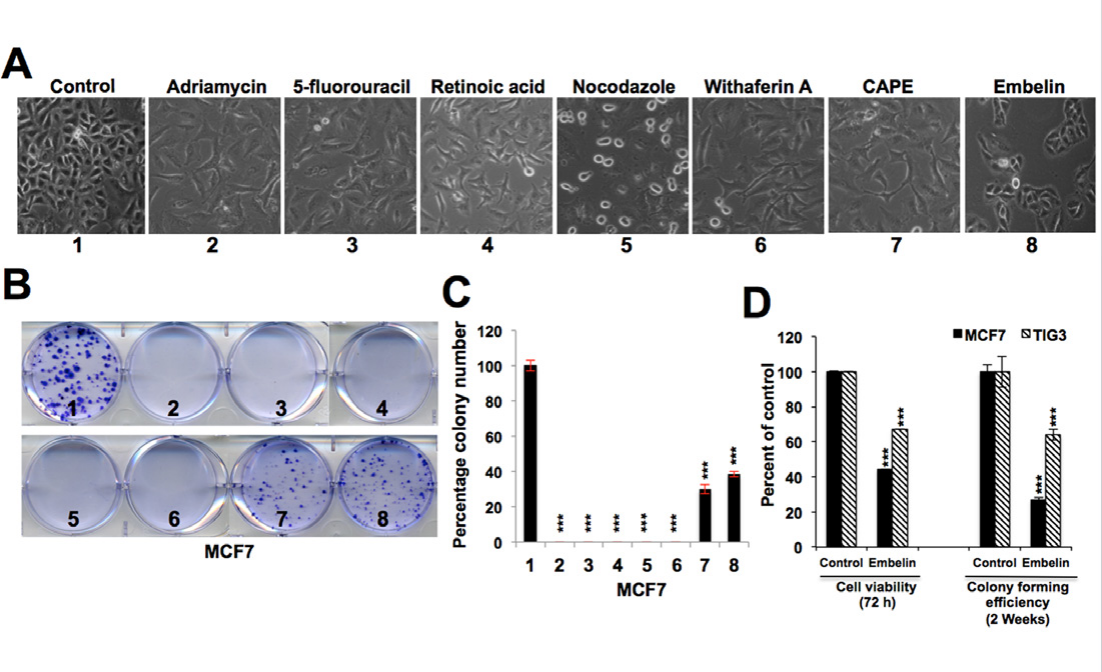
Comparison of the effect of anticancer drugs on short- and long-term proliferation of breast cancer cell lines. (**A**) Morphology of control and treated breast cancer cells indicative of growth arrest phenotype. (**B**) Colony formation assay of control and treated breast cancer cells (MCF7) showing decrease in treated cells. (**C**) Quantitation of the colony-forming assay from three independent experiments. (**D**) Comparative effect of embelin on cancer (MCF7) and normal (TIG-3) cells as detected by short term (viability) and long term (colony forming efficiency) growth assays. Statistical significance was calculated by QuickCals t-test calculator (***P<0.001).

**Fig 3:**
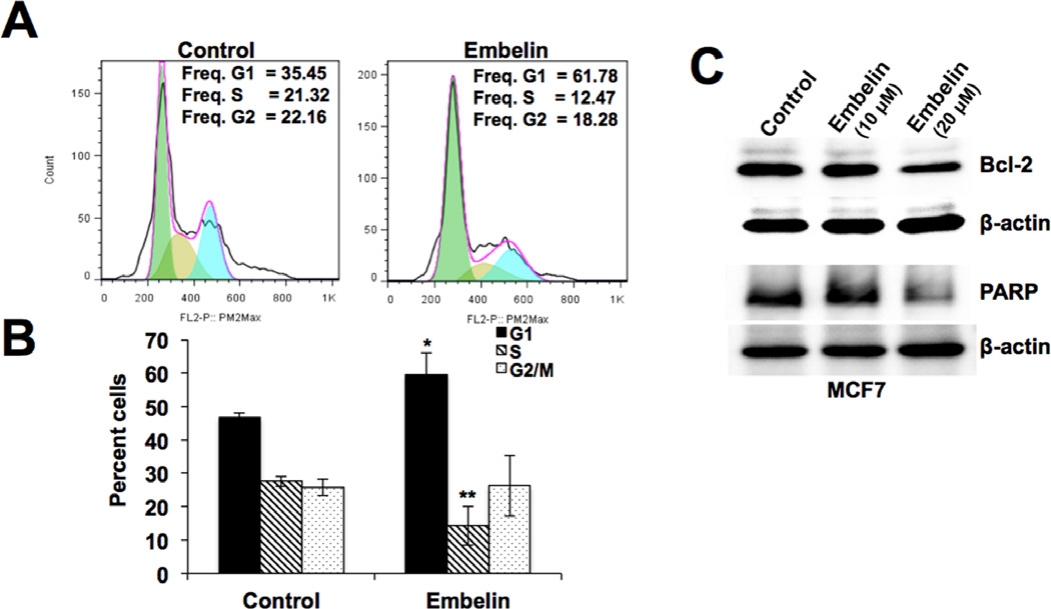
Determination of growth arrest versus apoptosis in response to treatment with embelin on MCF7 cells. (**A**) Cell cycle analysis of control and embelin (10 μM)-treated MCF7 cells indicating arrest of cells in G1 phase. (**B**) Histogram from three independent cell cycle experiments. Statistical significance was calculated using QuickCals t-test calculator (*P<0.05, **P<0.01, ***P<0.001). (**C**) Expression level of Bcl-2 and PARP proteins in control and embelin (10 μM and 20 μM)-treated MCF7 cells showing decrease in these anti-apoptotic proteins only in cells treated with 20 μM embelin. Actin was used as an experimental control.

Human breast cancer cells, MCF7 and MDA-MB-231 (MDA), were treated with low doses (Fig 1A) of eight drugs known to possess anticancer activity. The level of mortalin expression in control and treated cells was determined by Western blotting and immunostaining. As shown in Fig 1B and 1C (and data not shown), we found a variable level of decrease in mortalin expression in treated cells. Maximum decrease was obtained in response to caffeic acid phenethyl ester (CAPE) and embelin treatment in three independent experiments (Fig 1C). Immunostaining analyses further confirmed that most of these drugs caused decrease in mortalin in both the cell lines. Furthermore, natural drugs CAPE and embelin were as strong as adriamycin, an established anticancer drug in clinical use. Short-and long-term cell viability assays have earlier revealed that embelin caused strong cytotoxicity to cancer cells [10]. Furthermore, the effect of embelin at 10–20 μM concentration was comparable to other drugs that induce growth arrest of cells (Fig 2A). In long term colonogenic assays, embelin (10 μM) showed milder effect compared to other anticancer reagents, including adriamycin, fluorouracil, retinoic acid, nocodazole, withaferin A, and CAPE (Figs 1A, 2B and 2C). However, in contrast to other anticancer drugs that are extremely toxic to normal cells, embelin showed a gentle effect (Fig 2D) suggetsing that it may serve as a mild and safe anticancer reagent. Hence, further molecular analyses was warranted to elucidate the mechanism(s) of its action. In light of the above findings, we first examined the cell cycle distribution of control and embelin-treated MCF7 cultures. As shown in Fig 3A and 3B, embelin (10 μM) caused cell cycle arrest at G1 stage. Lack of apoptosis in cells treated with 10 μM embelin was confirmed by the absence of any change in the level of expression of proteins (Bcl-2, and Pro-PARP) involved in apoptotic signaling (Fig 3C). On the other hand, higher dose of embelin (20–30 μM) were seen to cause apoptosis,associated with decrease in Bcl-2 and Pro-PARP (Fig 3C and data not shown). Taken together, the data suggested that the low doses of embelin may target mortalin and cause growth arrest of cells by activation of tumor suppressor p53.

### Embelin docks into mortalin—p53 complex, abrogates their interaction and causes activation of p53

**Fig 4:**
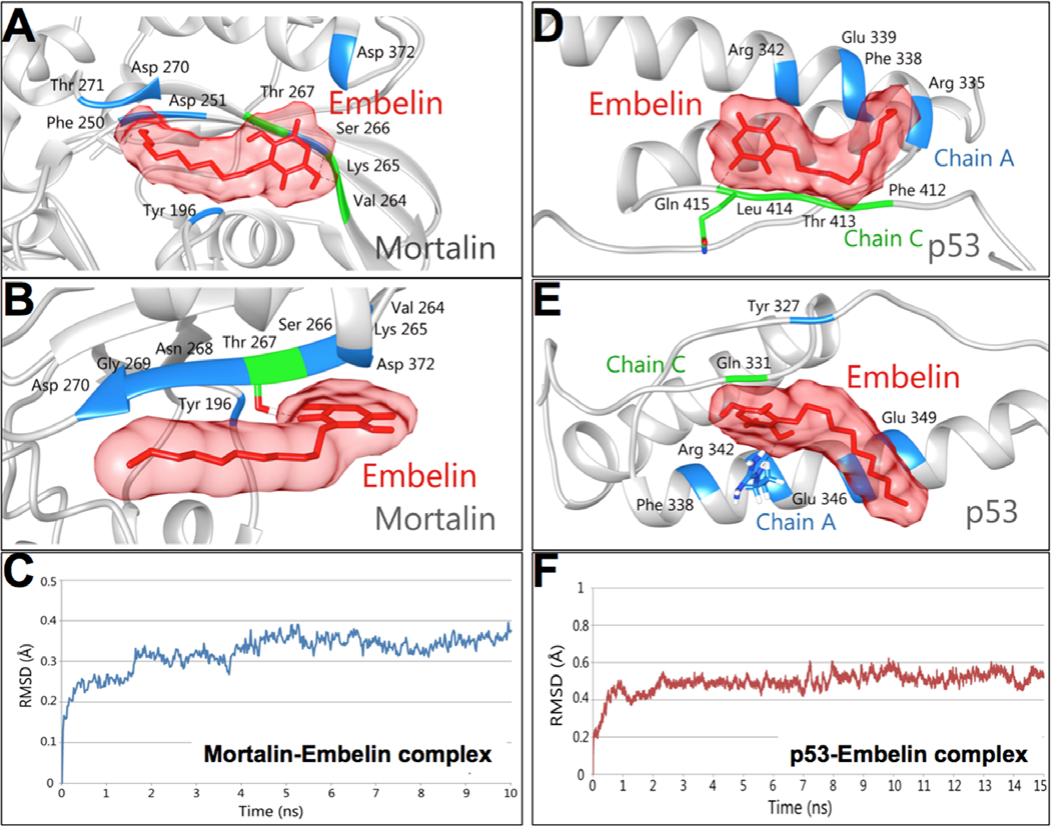
Molecular docking analysis of embelin with mortalin and p53. Hydrogen bond (green) and hydrophobic interactions (blue), before (**A**) and after (**B**) MD simulations of embelin-mortalin complex. (**C**) RMSD trajectory of Mortalin in complex with embelin after MD simulations. Hydrogen bond and hydrophobic interactions before (**D**) and after (**E**) MD simulations of embelin-p53 complex. (**F**) RMSD trajectory of p53 in complex with embelin after MD simulations.

**Fig 5:**
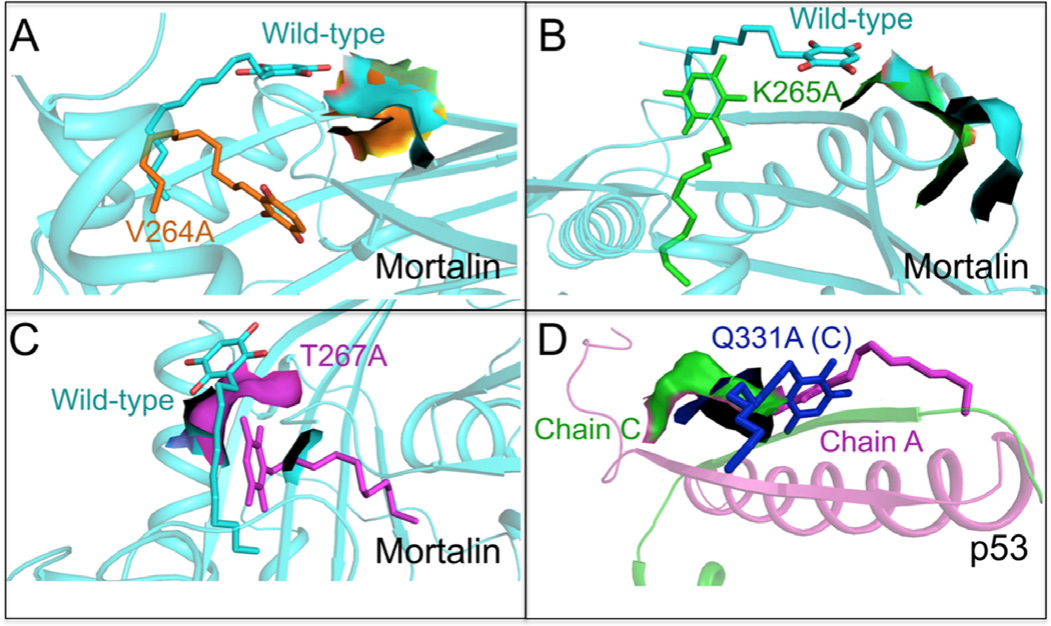
Molecular docking analysis of embelin with mutant mortalin and p53 proteins. The change in conformation of embelin and binding site in (**A**) wild type (light blue) and V264A (orange) mortalin (**B**) wild type (light blue) and K265A (green) mortalin (**C**) wild-type (light blue) and T267A (purple) mortalin and (**D**) wild type chain C (green) and Q331A chain C (blue) p53.

Mortalin has been shown to inactivate p53 protein [22, 30, 43–47]. In light of the above data, we next investigated the direct docking potential of embelin to mortalin and p53 proteins. Embelin was docked onto the crystal structures of mortalin and p53. The p53 binding site of mortalin (253 to 282) and the mortalin binding site of p53 (323 to 337) were used for carrying out docking studies. Embelin was observed to bind to mortalin with a docking score of -6.89 kcal/mol. Multiple hydrogen bonds and hydrophobic interactions, as shown in Fig 4A–4C, were observed in the docked complex. Embelin formed three hydrogen bonds with residues, Thr 267, Lys 265 and Val 264 of mortalin while residues Tyr 196, Phe 250, Asp 251, Ser 266, Asp 270, Thr 271 and Asp 372 of mortalin were involved in forming hydrophobic interactions with embelin. The third oxygen atom of embelin formed two hydrogen bonds with oxygen atom (O) of Val 264 (2.89 Å) and oxygen atom (O) of Lys 265 (2.91 Å). The third hydrogen bond was formed between first oxygen atom of embelin and oxygen atom of Thr 267 (2.70 Å). Together the hydrogen bonds and hydrophobic interactions formed a stable protein-ligand complex (Fig 4A and 4B). To further validate the importance of binding site, we mutated the three critical residues of mortalin involved in hydrogen bond formation (Val 264, Lys 265 and Thr 267) to Alanine and then performed docking. Mutation of these critical residues led to reduction in the binding affinity of embelin for mortalin. The binding scores exhibited by mutants V264A, K265A and T267A were -3.03 kcal/mol, -2.71 kcal/mol and -2.74 kcal/mol, respectively. The comparison of binding of embelin to the wild type mortalin and mutants is shown in Fig 5A–5C and Table 1. On the other hand, embelin was found to bind to Chain A and C of p53 with a high binding affinity of -9.97 kcal/mol. The ligand was observed to interact with the protein by forming a hydrogen bond and several hydrophobic interactions (Fig 4D–4F). The oxygen atom of Gln 331 (C) formed one hydrogen bond with third oxygen atom (2.74Å) of embelin. Residues Arg 335 (A), Phe 338 (A), Glu 339 (A), Arg 342 (A), Phe 328 (C), Thr 329 (C) and Leu 330 (C) of p53 were observed to be involved in forming hydrophobic interaction (Fig 4E). Embelin was found to form a stable complex with p53 embedding itself inside the cavity of p53 lined by mortalin binding residues clearly suggesting the non-formation of complex between mortalin and p53 (Fig 4D and 4E). The critical residue Gln 331 (C) involved in hydrogen bond formation with embelin was also mutated to Alanine to validate the drug binding site. A decrease in the binding affinity of mutant Q331A(C) with embelin (-3.91 kcal/mol) was observed in comparison to wild type (-9.97 kcal/mol). Comparison of binding of wild type and mutant p53 with embelin is shown in Fig 5D and S1 Table. The loss of hydrogen bonds and a substantial drop in the binding affinity of embelin for mutated structures of mortalin and p53 indicated the specificity of the molecular docking results. We also performed molecular dynamics simulations and energy stabilization of both wild type mortalin-embelin and p53-embelin complexes. Stability of embelin-docked mortalin structure was further analyzed by simulating the complex for 10 ns. The complex was observed to be stable for a period of 5 ns from 5 to 10 ns. The stabilization of the protein-ligand complex was evident from the stable RMSD trajectory (Fig 4C) that showed a little deviation during the first few nanoseconds but later got stabilized. Changes were observed in the interacting residues of mortalin as shown in Fig 4B. While there was only one hydrogen bond between oxygen atom of Thr 267 and oxygen atom of embelin, the residues involved in hydrophobic interactions increased post simulations. Residues Tyr 196, Val 264, Lys 265, Ser 266, Asn 268, Gly 269, Asp 270 and Asp 372 were found to be involved in hydrophobic interactions after simulations. Embelin-docked p53 complex was subjected to simulations for 15 ns. The docked complex was stable from 10 to 15 ns as can be seen from RMSD trajectory (Fig 4F). Few changes were observed in the interaction pattern of embelin with p53. The nitrogen atom of Arg 342 (A) formed a hydrogen bond of length 3.28Å with oxygen atom of embelin. Several residues Tyr 327 (A), Phe 338 (A), Glu 346 (A), Glu 349 (A) and Gln 331 (C) were involved in hydrophobic interaction with embelin (Fig 4E). Despite changes in the interaction pattern of ligand-docked protein complex, the structure was found more stable after simulations.

**Fig 6:**
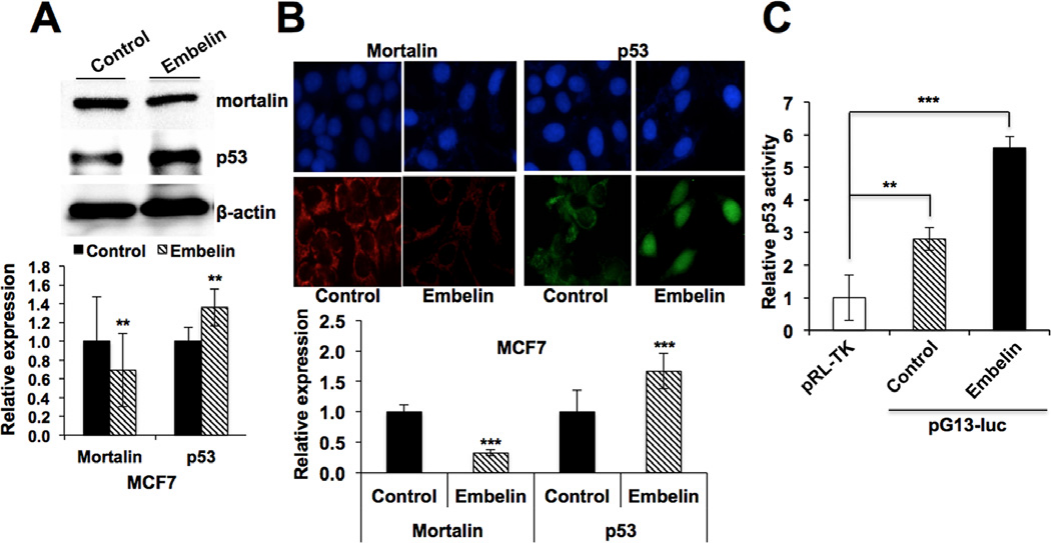
Embelin treatment downregulates mortalin expression and enhances the nuclear localization and transcriptional activation of p53. (**A**) Mortalin and p53 expression levels in control and embelin-treated breast cancer (MCF7) cells, as detected by Western blotting. Actin was used as an internal control. Quantitation of protein expression levels from three independent experiments performed using ImageJ software is shown in the lower panel. (**B**) Immunostaining of mortalin and p53 in control and embelin (10 μM)-treated (MCF7) cells showing reduction in mortalin level and increased in nuclear localization of p53 in response to embelin treatment. Intensity measurement of immunostaining was done by ImageJ software. (**C**) Relative p53 activity in control and embelin-treated cells was determined by p53-dependent luciferase reporter assay. Statistical significance was calculated using QuickCals t-test calculator (*P<0.05, **P<0.01, ***P<0.001).

In light of the above data on the decrease of mortalin in embelin-treated cells and bioinformatics predictions, we investigated the expression of p53 and its activity were determined by Western blotting, immunostaining and reporter assays. As shown in Fig 6A and data not shown, there was an increase in p53 in embelin-treated cells that showed decrease in mortalin. Immunostaining revealed the nuclear translocation of p53 (Fig 6B). Transcriptional activation of p53 in embelin-treated cells was confirmed by p53-dependent reporter assays (Fig 6C).

### Embelin downregulates mortalin as well as metastatic signalling

**Fig 7:**
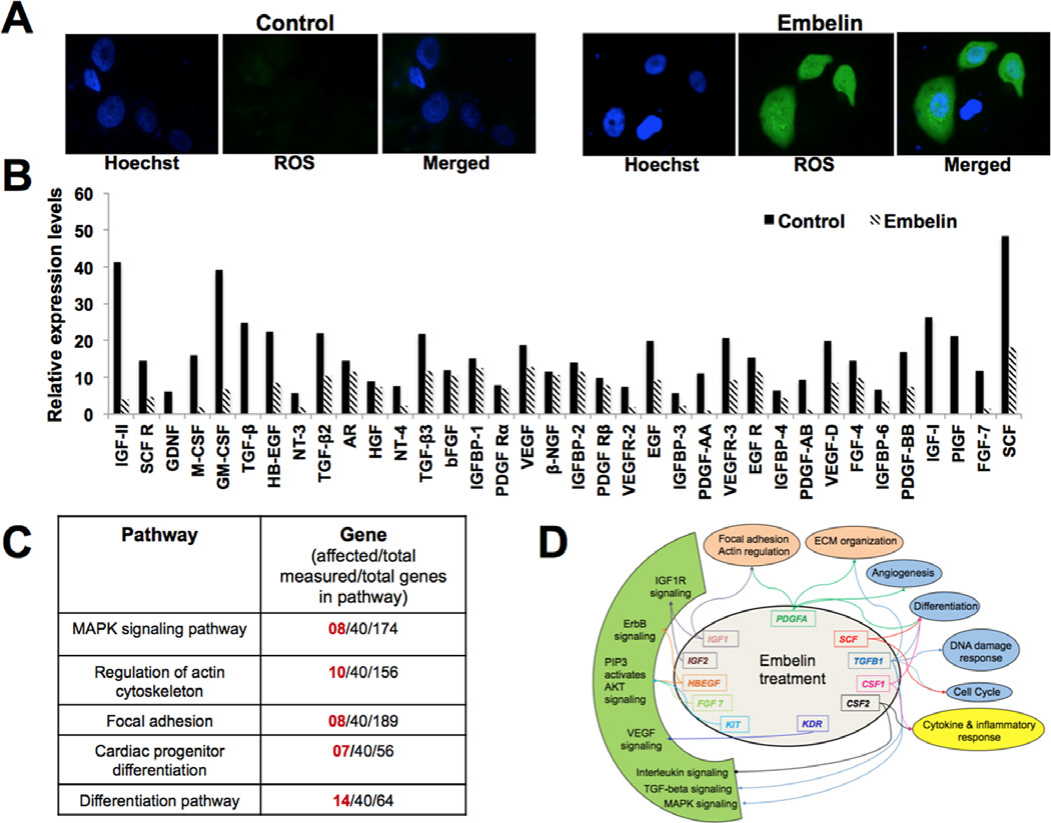
Effect of embelin on ROS level and metastasis regulating proteins. (**A**) ROS staining in control and embelin-treated cells is shown. (**B**) Relative expression levels of secreted proteins in control and embelin (10 μM)-treated breast cancer (MCF7) cells, as obtained by antibody membrane array, depicting decrease in secreted protein levels after embelin treatment. (**C**) List of pathways regulated by proteins shown in **B**. (**D**) Graphical representation of pathways and genes affected by embelin treatment. Proteins shown in the inner circle were downregulated in embelin-treated cells. Pathways regulated by these proteins are indicated in the outer circle. Colored arrows are used to link genes with associated pathways.

**Fig 8:**
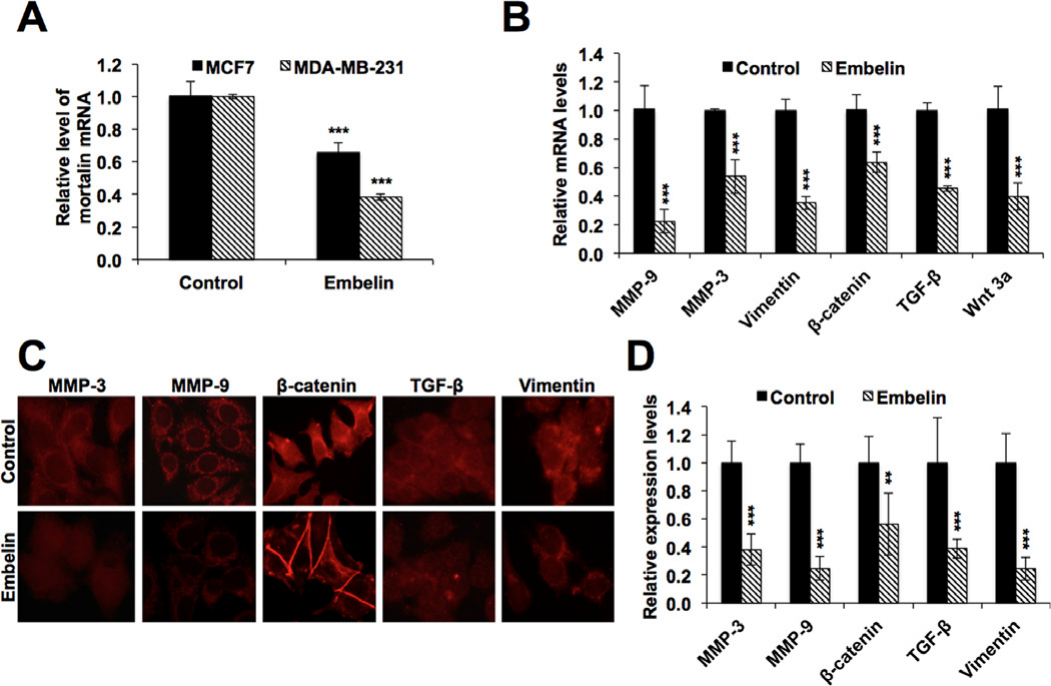
Effect of embelin on mRNA and protein levels of mortalin and other metastasis-regulatory genes. (**A**) mRNA levels of mortalin, as obtained by quatitative real time PCR, in control and embelin (10 μM)-treated cellsshowing transcriptional downregulation of mortalin in treated cells, obtained by quantitative real time PCR experiment. (**B**) mRNA levels of proteins involved in metastasis in control and embelin (10 μM)-treated (MCF7) cells showing their transcriptional downregulation in embelin-treated cells. (**C**) Immunostaining of metastatic regulatory proteins in control and embelin-treated cells (MCF7). (**D**) Intensity measurement of immunostaining shown in (C) was done by ImageJ software. Statistical significance was calculated using QuickCals t-test calculator (*P<0.05, **P<0.01, ***P<0.001).

Mortalin has been shown to regulate ROS level [47, 48] and promote metastasis of cancer cells [22, 47, 49]. We examined the level of ROS in control and embelin-treated cells, and found that it to be significantly higher in the treated cells (Fig 7A). We next examined the status of metastatic signaling in control and embelin-treated cells by antibody membrane array. As shown in Fig 7B, embelin caused reduction in many proteins involved in metastatic signaling. Pathway analysis of these proteins revealed the involvement of several major pathways including MAPK signaling, regulation of actin cytoskeleton, focal adhesion, ECM organization, angiogenesis, DNA damage response, cell cycle regulation, cytokine and inflammatory response and differentiation (Fig 7C and 7D). We found that embelin caused significant decrease in the expression of TGF-β1, PDGF-A, IGF-1, IGF-II, KIT, KDR, CSF2 and PIGF proteins, involved in numerous biological pathways regulating cell proliferation and differentiation characteristics of cells (Fig 7C and 7D). Besides, embelin also downregulated the growth factors and proteins involved in regulation of cytoskeleton, cell adhesion and extracellular matrix organization (S2 Table). In order to further determine if these changes were the result of decreased level of expression in mortalin and metastatic proteins, we performed quantitative PCR with gene specific primers. As shown in Fig 8A–8D, there was a significant downregulation of mRNA levels of mortalin and several other proteins, including MMPs, vimentin, β-catenin, TGF-β and Wnt-3a, involved in cancer cell metastasis in embelin-treated cells as compared to their control counterparts. Immunostaining confirmed these changes at the protein level (Fig 8C and 8D).

## Discussion

Several studies have reported that embelin causes apoptosis in a dose- and time-dependent manner [20]. It has been shown to cause changes in mitochondrial membrane potential [20], downregulate XIAP (X-chromosome linked inhibitor of apoptosis) and a short isoform of FLIP leading to TRAIL (Tumor necrosis factor-related apoptosis-inducing ligand)-induced apoptosis [50–53]. Recent studies have indicated that the mitochondria and lysosomes are prominent targets of embelin-induced cell death response, where it leads to loss of mitochondrial membrane potential [20, 54]. Mortalin is an essential protein as it regulates mitochondrial-genesis, membrane potential and energy generation processes [55]. Cells compromised for mortalin undergoes growth arrest and mitochondrial fragmentation suggesting that it is indeed an essential protein for mitochondrial integrity. Decrease in mortalin in embelin-treated cells was associated with increase in tumor suppressor p53 and ROS that attributes, at least in part, to the growth arrest of cells. We further demonstrate that embelin could potentially abrogate mortalin-p53 complexes, resulting in the nuclear translocation of p53 (as observed by immunostaining) and activation of its transcriptional activation function (as observed by p53-dependent reporter assay). In addition to abrogation of mortalin-p53 complexes by embelin, decrease in mortalin was observed both at the mRNA and protein levels in embelin-treated cells. Such decrease in mortalin was anticipated to affect important functions of mortalin including regulation of ROS and cancer cells metastasis [47–50]. We found that, indeed, embelin-treated cells possess higher level of ROS and low level of metastasis-regulatory proteins (MMPs, vimentin, β-catenin, TGF-β and Wnt-3a) suggesting that these may be mediated, at least in part, by targeting mortalin by embelin. *In vivo* study performed on Wistar rats, Albino rats and mice have depicted significant anti-tumor potential of embelin, wherein it was shown to enhance the survival of treated animals [56–58]. Taken together, we found that embelin targets an essential chaperone, mortalin. We demonstrate that the downregulation of mortalin resulting in activation of p53 and ROS signaling, and deactivation of metastasis signaling is one of the mechanisms involved in the anticancer activity of embelin.

## Supporting Information

S1 Table Interaction pattern of embelin with mutated mortalin and p53. (DOCX)

S2 Table Effect of embelin on metastasis regulatory proteins (relative units of expression) and pathways. (DOCX)
