# Supplementary material for: Targeting Mortalin by Embelin Causes Activation of Tumor Suppressor p53 and Deactivation of Metastatic Signaling in Human Breast Cancer Cells
Source: PLoS One. 2015 Sep 16;10(9):e0138192. doi: 10.1371/journal.pone.0138192 (PMC4574062; doi:10.1371/journal.pone.0138192)
Supplement: S1 Table — (DOCX) [file pone.0138192.s001.docx]

**S1 Table.** Interaction pattern of embelin with mutated mortalin and p53.

| **Complex** | **Mutant** | **Residues involved in** | |
| --- | --- | --- | --- |
|  |  | **Hydrogen bond formation** | **Hydrophobic interaction** |
| Mortalin-embelin | V264A | - | Val 193, Pro 194, Glu 222, Pro 223, Thr 249, Phe 250, Asp 251, Ile 252, Ser 253, Thr 267, Tyr 196, Asn 268, Gly 269, Asp 270, Thr 271 |
|  | K265A | - | Pro 194, Ala 195, Tyr 196, Pro 223, Glu 222, Ala, 226, Asp 244, Asp 251, Ser 253, Thr 267, Asn 268, Val 386, Val 417 |
|  | T267A | - | Pro 194, Ala 195, Tyr 196, Glu 222, Pro 223, Val 242, Asp 244, Asp 251, Ile 252, Ser 253, Ala 267, Asn 268, Val 386, Val 417 |
| p53-embelin | Q331A(C) | - | Phe 328 (A), Thr 329 (A), Leu 330 (A), Gln 331 (A), Phe 338 (A), Arg 342 (A), Asn 345 (A), Thr 329 (C), Leu 330 (C), Ala 331 (C) |
