# Supplementary material for: Targeting Mortalin by Embelin Causes Activation of Tumor Suppressor p53 and Deactivation of Metastatic Signaling in Human Breast Cancer Cells
Source: PLoS One. 2015 Sep 16;10(9):e0138192. doi: 10.1371/journal.pone.0138192 (PMC4574062; doi:10.1371/journal.pone.0138192)
Supplement: S2 Table — (DOCX) [file pone.0138192.s002.docx]

**S2 Table.** Effect of embelin on metastasis regulatory proteins (relative units of expression) and pathways.

| **Pathway name** | **Affected/Total genes** | **Gene Name** | **Control** | **Embelin** |
| --- | --- | --- | --- | --- |
| **Differentiation pathway** | 14/64 | TGFB1 | 24.85 | 0 |
|  |  | IGF-I | 26.21 | 0 |
|  |  | EGF | 19.90 | 9.44 |
|  |  | PDGFA | 11.09 | 0.99 |
|  |  | VEGFA | 18.76 | 12.72 |
|  |  | FGF-4 | 14.48 | 9.82 |
|  |  | SCF | 48.00 | 18.05 |
|  |  | CSF1 | 11.98 | 1.89 |
|  |  | KIT | 14.53 | 4.69 |
|  |  | TGF-β3 | 21.78 | 11.67 |
| **MAPK signaling pathway** | 8/174 | NTF5 | 7.68 | 2.25 |
|  |  | EGF | 19.9 | 9.44 |
|  |  | EGFR | 15.28 | 11.42 |
|  |  | FGF-4 | 14.46 | 9.82 |
|  |  | PDGF8 | 16.85 | 7.5 |
|  |  | TGFB1 | 24.8 | 0 |
|  |  | TGF-β2 | 21.99 | 10.52 |
|  |  | TGF-β3 | 21.78 | 10.52 |
| **Focal adhesion** | 8/189 | PDGFA | 11.09 | 0.99 |
|  |  | HGF | 28 | 0 |
|  |  | EGF | 19.9 | 9.44 |
|  |  | FIGF | 19.82 | 8.46 |
|  |  | PDGFB | 16.85 | 7.5 |
|  |  | EGFR | 15.28 | 11.42 |
|  |  | KDR | 7.42 | 1.84 |
|  |  | IGF-I | 26.21 | 0 |
| **PIP3 activates AKT signaling** | 6/172 | HBEGF | 22.42 | 8.43 |
|  |  | EGF | 19.9 | 9.44 |
|  |  | FGF-4 | 9.87 | 14.48 |
|  |  | FGF-7 | 1.46 | 11.65 |
|  |  | KIT | 4.69 | 14.53 |
|  |  | EGFR | 15.28 | 11.42 |
| **Regulation of actin cytoskeleton** | 5/156 | PDGFA | 11.09 | 0.99 |
|  |  | PDGFB | 16.85 | 7.5 |
|  |  | EGF | 19.9 | 9.44 |
|  |  | EGFR | 15.28 | 11.42 |
|  |  | FGF-4 | 14.48 | 9.82 |
| **Cardiac progenitor differentiation** | 7/56 | TGF-β1 | 24.85 | 0 |
|  |  | IGF-I | 26.21 | 10.28 |
|  |  | IGF-II | 41.23 | 4.03 |
|  |  | KDR | 7.42 | 1.84 |
|  |  | KIT | 14.53 | 4.69 |
